# Unified Mobile App for Streamlining Verbal Autopsy and Cause of Death Assignment in India: Design and Development Study

**DOI:** 10.2196/59937

**Published:** 2025-01-10

**Authors:** Harleen Kaur, Stuti Tripathi, Manjeet Singh Chalga, Sudhir K Benara, Amit Dhiman, Shefali Gupta, Saritha Nair, Geetha Menon, B K Gulati, Sandeep Sharma, Saurabh Sharma

**Affiliations:** 1 ICMR-National Institute for Research in Digital Health and Data Science, Ansari Nagar, New Delhi, 110029, India, 91 7840870009; 2 Indian Council of Medical Research, New Delhi, India; 3 ICMR-National JALMA Institute for Leprosy & Other Mycobacterial Diseases, Agra, India

**Keywords:** verbal autopsy, cause of death, mortality, mHealth, public health, India, mobile health

## Abstract

**Background:**

Verbal autopsy (VA) has been a crucial tool in ascertaining population-level cause of death (COD) estimates, specifically in countries where medical certification of COD is relatively limited. The World Health Organization has released an updated instrument (Verbal Autopsy Instrument 2022) that supports electronic data collection methods along with analytical software for assigning COD. This questionnaire encompasses the primary signs and symptoms associated with prevalent diseases across all age groups. Traditional methods have primarily involved paper-based questionnaires and physician-coded approaches for COD assignment, which is time-consuming and resource-intensive. Although computer-coded algorithms have advanced the COD assignment process, data collection in densely populated countries like India remains a logistical challenge.

**Objective:**

This study aimed to develop an Android-based mobile app specifically tailored for streamlining VA data collection by leveraging the existing Indian public health workforce. The app has been designed to integrate real-time data collection by frontline health workers and seamless data transmission and digital reporting of COD by physicians. This process aimed to enhance the efficiency and accuracy of COD assignment through VA.

**Methods:**

The app was developed using Android Studio, the primary integrated development environment for developing Android apps using Java. The front-end interface was developed using XML, while SQLite and MySQL were employed to streamline complete data storage on the local and server databases, respectively. The communication between the app and the server was facilitated through a PHP application programming interface to synchronize data from the local to the server database. The complete prototype was specifically built to reduce manual intervention and automate VA data collection.

**Results:**

The app was developed to align with the current Indian public health system for district-level COD estimation. By leveraging this mobile app, the average duration required for VA data collection to ascertainment of COD, which typically ranges from 6 to 8 months, is expected to decrease by approximately 80%, reducing it to about 1‐2 months. Based on annual caseload projections, the smallest administrative public health unit, health and wellness centers, is anticipated to handle 35‐40 VA cases annually, while medical officers at primary health centers are projected to manage 150‐200 physician-certified VAs each year. The app’s data collection and transmission efficiency were further improved based on feedback from user and subject area experts.

**Conclusions:**

The development of a unified mobile app could streamline the VA process, enabling the generation of accurate national and subnational COD estimates. This mobile app can be further piloted and scaled to different regions to integrate the automated VA model into the existing public health system for generating comprehensive mortality statistics in India.

## Introduction

Effective and efficient public health surveillance systems rely heavily on communication, collaboration, and information sharing. Improving traditional data collection and reporting methods is essential for supporting surveillance and response efforts. Cause-specific mortality is an important component in planning and implementing public health interventions. Digital technologies, especially in India, have been instrumental in achieving these goals while advancing the vision of transforming into a digitally empowered society. This revolution has accelerated the growth of health care services. These measures enable public health professionals across various regions to collaborate and communicate effectively while gathering crucial information efficiently, thereby facilitating rapid and targeted interventions by generating and disseminating public health information [1].

Cause of death (COD) information is essential for evaluating health interventions and programs at all levels. Ideally, civil registration systems provide relevant information related to births, deaths, and COD in any country [2-4]. However, in countries with a large population such as India, obtaining comprehensive and reliable information on COD has been a major challenge. The death registration rates in India have shown considerable progress with approximately 93% of estimated deaths officially registered. However, only 19% of these registered deaths have a medically certified cause of death (MCCD) [5]. To address this gap, research studies have suggested that detailed household interviews with family members of the deceased, known as verbal autopsy (VA), can improve the quality of information on COD [6-8]. The primary aim of VA is to ascertain population-level COD estimates. In a VA, the COD is ascertained based on an interview with next of kin or other caregivers using a standardized questionnaire that gathers information on signs, symptoms, medical history, and circumstances preceding death [9]. Data collected during these interviews is then processed and analyzed to assign a probable COD. Standard VA instruments include a VA questionnaire, a list of COD, and diagnostic criteria (based on either expert or data-driven algorithms) for assigning a COD. The World Health Organization’s (WHO) VA instrument contains a concise list of COD that are of public health importance. These can be determined through a significant number of questions designed for use in VA interviews and with automated or physician-coded COD assignment using analytical software. The Office of the Registrar General of India has been successfully conducting VA since 1999, with the help of trained physicians to assign COD [4,10].

The existing WHO VA Instrument is compatible with the Open Data Kit (ODK) Android app and has been tested for feasibility in the Indian context [11]. The ODK app is designed to be used in various scenarios such as surveillance, surveys, research, and crisis response. The current ODK app provides diverse features such as reducing data entry costs and preventing missing or invalid data from VA questionnaires. However, the development of mobile apps often stems from specific requirements for national-level VA studies and surveillance programs. This study by the Indian Council of Medical Research–National Institute for Research in Digital Health and Data Science (ICMR-NIRDHDS) details the development of a unified mobile app to streamline the data collection process during VA by using the existing public health workforce. This app is designed to support the line listing of death records, with further segregation of data by user and field catchment area, which enhances scalability and efficiency in data management. As the volume of cases increases, integration of these capabilities directly into the app helps prevent bottlenecks in data collection and analysis, streamlining the entire process. Additionally, the app includes automated data transfer for physician-coded verbal autopsy (PCVA), reducing the need for manual data extraction and sharing, which accelerates data processing and minimizes delays. It could facilitate streamlining of the data collection process and enable physicians to assign COD within a single app, completely integrated with the public health system from the subcenters to district hospitals.

## Methods

### Study Design

**Figure 1.**
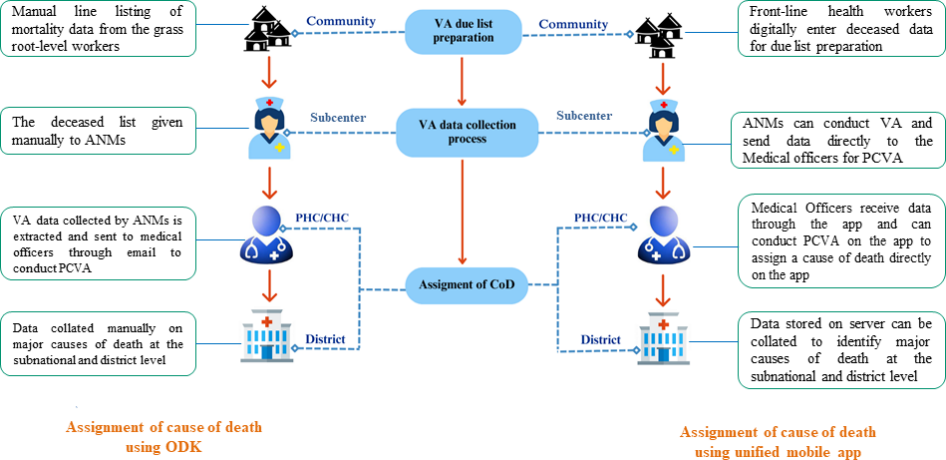
Step-by-step workflow for cause of death assignment using the unified mobile app, highlighting data collection, verification, and physician review. ANM: auxiliary nursing midwife; CHC: community health care; CoD: cause of death; ODK: Open Data Kit; PCVA: physician-coded verbal autopsy; PHC: primary health care; VA: verbal autopsy.

The WHO’s VA instrument was tested for its feasibility in the Indian context by using the computer-assisted personal interviewing device-based data collection method. The ICMR-NIRDHDS has thus taken the initiative to develop a mobile app to streamline VA data collection, which may be integrated into the existing public health system. A graphical example depicting the comparison of the existing ODK-based VA tool and the developed unified mobile app is shown in Figure 1.

### Phase 1: Ideation, Planning

Reliable cause-specific mortality statistics are required by administrators, policy planners, and researchers for evidence-based decision-making regularly. Assigning COD using PCVA and computer-coded VA methods has already been tested in urban as well as rural areas of India using the existing ODK app [11]. However, this approach involved manual intervention at various stages of the data collection process. Initial automated line listing of death records by accredited social health activist (ASHA) workers is not supported by the ODK and is instead maintained manually, followed by subsequent VAs conducted by auxiliary nursing midwives (ANMs), community health officers, and field investigators. Data collected during the VA process was then extracted from the ODK platform and transferred via email to medical officers for COD assignment. Manual interventions requiring an extensive workforce in large-scale studies are time-consuming and can potentially compromise the data quality and subsequent outcomes. Therefore, this study aimed to develop a unified app for eliminating the manual interventions at each stage of VA data collection and COD assignment, from death reporting to conducting PCVA in rural settings. Data collected using mobile or handheld devices such as computer-assisted personal interviewing tablets could also be transferred electronically to physicians, enabling them to assign COD efficiently. In addition to providing community-based perspectives on rural mortality, it also served as a basis for testing VA methods on an operational level. This app also offers offline data collection without an internet connection and limited resources. Hence, the primary goal of the study was to efficiently implement the WHO VA 2022 instrument in rural settings through the existing public health system, with plans for further expansion to integrate automated algorithms for assigning COD.

### Phase 2: App Development

**Figure 2.**
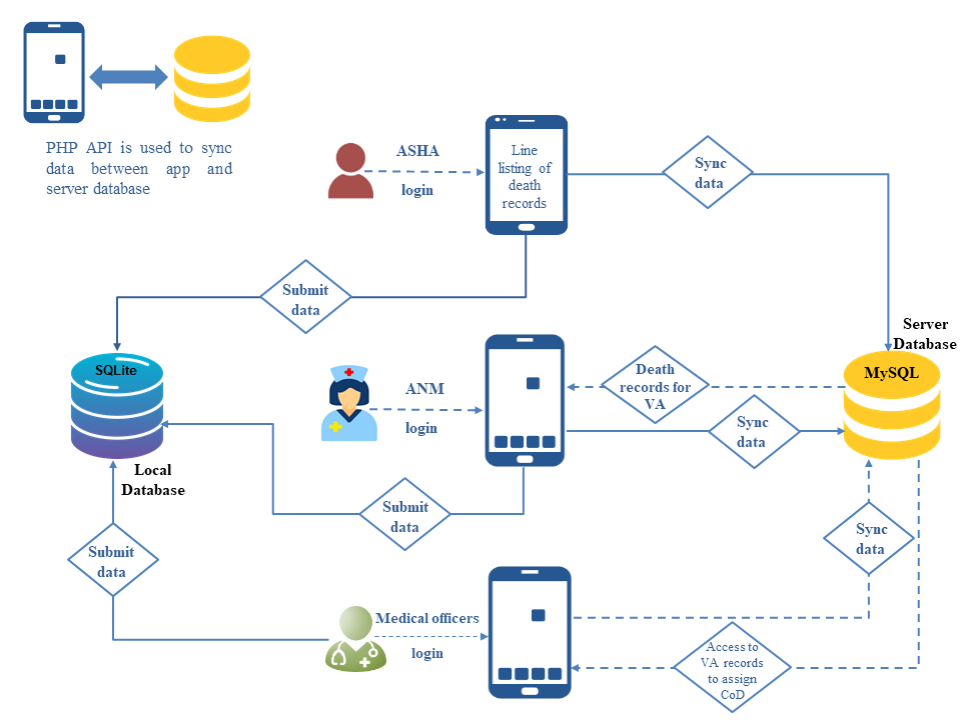
Diagram depicting the step-by-step data flow from user input in the mobile app to the central database, highlighting the distinct pathways for different user roles. ANM: auxiliary nursing midwife; API: application programming interface; ASHA: accredited social health worker; CoD: cause of death; VA: verbal autopsy.

**Figure 3.**
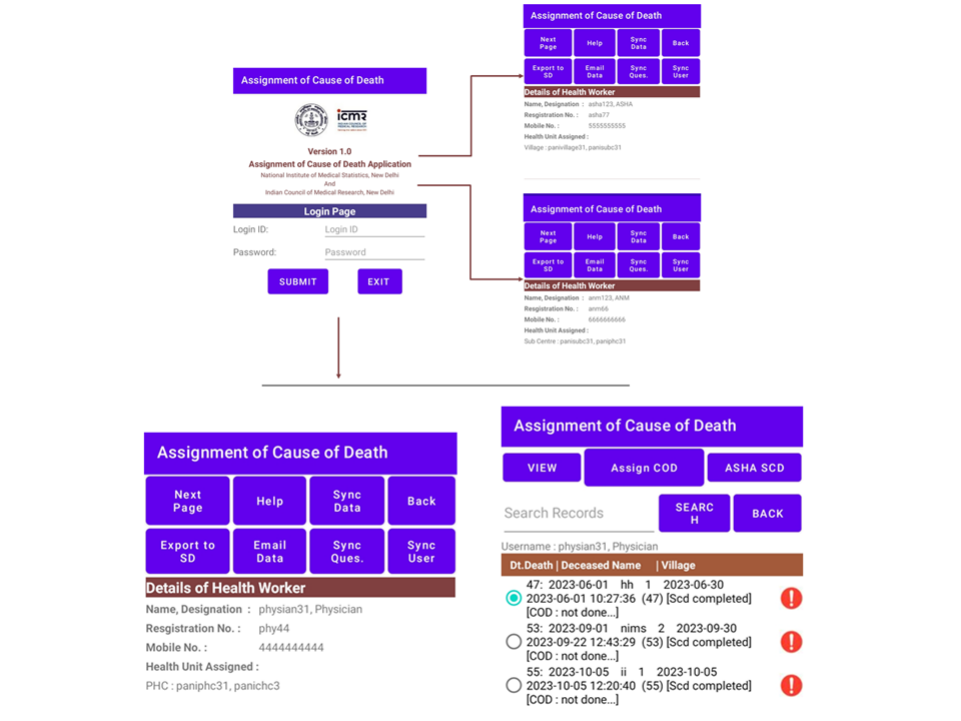
Customized app interface designed for specific user roles to streamline tasks for accredited social health workers, auxiliary nursing midwives, and medical officers.

Having conceptualized the solution, the next step was the development of the VA app by the team of ICMR-NIRDHDS. It was developed using Android Studio, which is the primary IDE for developing Android apps. This app was based on the WHO 2022 VA questionnaire comprising three modules, for example, adult, children, and neonate (data elements’ details are given in Multimedia Appendix 1). The entire app was developed using Java for programming and XML for designing the front-end interface. Data storage was streamlined using the MySQL database management system on a secure and authenticated server, while SQLite was used for local data storage within the app. The initial submission of data by each user is stored on the local database. Next, the user selects the Sync button to update data on the centralized server. Communication between the app and server was facilitated through a PHP application programming interface (API). The API acts as a bridge between the mobile app and the server to facilitate data synchronization. During this process, the app sends HTTP requests to the server using specific PHP API end points. These end points were designed to receive the submitted data, perform validation and sanitization, and subsequently insert or update the records in the MySQL server database. After data submission, the server response is sent back to the mobile app to update the corresponding local database records. Given the data complexity, we opted for a relational database model for comprehensive data analysis and to ensure structured data organization and efficient retrieval. Further, the developed app allowed the data collectors to partially complete the surveys and resume them at a later stage or edit responses after the initial submission of the survey. This facilitated standardized data collection for subsequent review by physicians involved in the study. The complete prototype was designed with the ability to capture data with minimal cognitive effort. The navigational structure depicting the complete network is shown in Figure 2. Snapshots of the user-based interface are shown in Figure 3.

### Phase 3: Data Management, Testing, and Quality Assurance

In this phase, the mobile app underwent field testing in both English and Hindi as an integral part of the methodology. Using APIs and servers for data communication ensured effective and secure data management, maintaining data integrity. Multiple data validation checks, skip patterns, and quality assurance measures were incorporated into the survey to maintain the accuracy and integrity of the data.

### Ethical Considerations

The study was reviewed and approved by the ICMR-NIMS Ethics Committee on Health Research (ICMR_NIMS_2020). Informed consent was obtained from all subjects involved in the study. Data security and patient privacy were prioritized throughout the entire process. The app adheres to strict security standards, with access controls and user authentication. Patient identifiers are anonymized to protect their identities. Subject compensation is not applicable in the present study and hence was not provided.

## Results

### Overview

The mobile app was developed with a focus on the integration and implementation of VA within the existing Indian public health system. Health and wellness centers, which serve approximately 5000 individuals, were projected to manage around 35‐40 VA cases annually, translating to about 3‐4 cases per month per center. The ANMs and community health officers, who are the primary health care providers in these centers, are tasked with performing and recording these autopsies efficiently using the app. Similarly, primary health centers catering to larger populations of around 20,000‐30,000 individuals are projected to handle a higher annual caseload of 150‐200 VAs, equating to 12‐16 autopsies per month per center for each medical officer. These projections were based on population densities and historical data on mortality rates in these regions, allowing for more accurate workload estimation. The app was specifically designed with these projections in mind, optimizing the workflow to manage these volumes with ease.

The traditionally lengthy process of VA data collection and COD determination, which generally spans 6‐8 months, is anticipated to be significantly expedited with this app, reducing this duration by approximately 80%. This would shorten the time from VA data collection to final COD determination to approximately 1‐2 months.

To ensure the app’s practicality and usability, extensive feedback was gathered from subject matter experts, including public health officials and field workers. This input resulted in several enhancements, such as simplifying data input processes and ensuring compatibility with existing health information systems.

### Prototype Evaluation: Experts’ Insights and Recommendations

The unified mobile app used in this study was developed according to the outlined objectives and methodologies. It was critically reviewed by the subject experts and its contents were refined according to their feedback and suggestions until final approval. Therefore, the developed app has automated the VA process and has been rated as efficient, user-friendly, and easy to navigate by the users and various stakeholders, strengthening the existing public health system. Textbox 1 lists the recommendations by the subject experts.

Textbox 1. **Experts’ recommendations implemented in the app.**

**Content**

- Customization of the user interface for ease of use by frontline health workers
- Addition of detailed information of the health care workers along with the demarcated health care centers, enabling the generation of an area-wise due list for conducting the verbal autopsy (VA)
- Incorporation of the list of cause of death with VA codes, which can be assigned by medical officers

**Interaction and workflow pathways**

- Streamlining of workflow pathways for frontline workers (eg, accredited social health activist [ASHA], auxiliary nursing midwife [ANM]) and medical officers
- Transition from manual line listing to digital due list preparation of deceased data
- Addressing the language barrier by facilitating bilingual VA process in both English and Hindi
- Creation of a user-friendly interface with role-based access defined for each user group (eg, ANM, ASHA, medical officers)

### App Overview

Figure 3 illustrates the app interface, beginning with the login module accessible only to authorized users. Role-based login credentials for specific roles such as ANM, ASHA, and medical officers will allow them to navigate to specific sections on the app. ASHA workers will be able to digitally enter the deceased data according to the demarcated health centers. This digitized data collection marks a significant shift from traditional manual methods, ensuring accuracy and minimizing errors.

This stored data will be further accessible to ANMs to conduct a VA using a bilingual interface (in Hindi and English), according to their feasibility. The VA process allows for partial saving of information, which can be completed at a later time—an important feature for real-world scenarios where time constraints are often encountered. Once ANMs sync the VA data, it becomes available for medical officers to review and assign a COD, facilitated by the integration of VA codes directly in the app.

The app’s robust design, which is grounded in expert feedback, facilitates an integrated and real-time approach to VA and COD assignment.

## Discussion

Overall death registration rates have shown significant improvement in India. However, the MCCD currently covers only a smaller percentage of deaths compared to the total registered deaths [12-14]. At present, the Office of the Registrar General of India conducts VA for a sample of deaths through the Sample Registration System survey [15]. VA holds significant potential to supplement MCCD and strengthen the mortality statistics at the population level [16-18]. In the conventional system, VA data collection involves a multistep, manual process where health workers fill out paper-based questionnaires and rely on physicians to review and assign causes of death, often leading to substantial delays.

The existing WHO VA instrument is compatible with the ODK software and has undergone feasibility testing in the Indian context. However, this app is primarily designed for conducting VA and requires manual intervention at each stage, which limits its flexibility and adaptability across different states. To scale up the existing VA survey, it could be integrated with the existing public health system through the grassroots-level workforces and digital health technology [9,19]. Over the past decade, the role of digital health technologies in public health has been increasing [20]. In India, several digital health initiatives, such as doctor consultation apps, e-pharmacies, and e-Sanjeevani, were developed to strengthen the existing public health system [21-24]. As an advancement, the institute has developed a unified mobile app to streamline the COD assignment process via VA. By utilizing the public health system, this mobile app for VA can act as a stepping stone toward addressing the challenge of timely and accessible mortality data collection in India. With the increasing penetration of mobile devices across urban and rural areas, this app has the potential to reach diverse populations, including those in remote regions. This study was able to demonstrate that a customized smartphone app for VA data collection, data transfer, and COD identification can be used for disease burden estimation at subpopulation levels. The app is further scalable for use in larger populations, including urban regions and more tertiary hospitals. Additionally, India is characterized by its cultural and linguistic diversity, presenting a challenge in obtaining accurate VA data. The app can further accommodate multiple languages and aid in overcoming this barrier.

The mobile app developed comes with several key features and strengths: (1) it assists inline listing of the cases and due list preparation; (2) frontline workers can conduct VA through this mobile app; (3) VA cases can be automatically assigned based on the field catchment area; (4) physicians can retrieve information from the server and conduct PCVA; (5) it is bilingual, supporting both English and Hindi; (6) it has the potential to substantially improve the timeliness of mortality data reporting at both the local and national levels by reducing the overall time from 6 to 8 months to just 1‐2 months; (7) the mobile app can be customized based on the variability of public health organizational structure across different states; (8) the app could accommodate multiple languages; (9) the app could be functional in remote areas with limited internet connectivity, as it allows users to transfer and retrieve data from the server offline; and (10) health care workers can retrieve data from the server at any point of time, enhancing the timely accessibility of the information.

However, mobile health implementation is often encountered with challenges [25,26]. Acquiring and maintaining hardware is difficult due to financial limitations and inadequate technical expertise. Developing the complete workflow from data collection to assigning the COD for each user was also challenging initially. However, it improved gradually through multiple discussions with various stakeholders. Additionally, Android apps may face compatibility issues across different devices and operating system versions, leading to inconsistent performance and user experience.

In conclusion, the development of a unified mobile app for streamlining VA and COD assignment through the public health system in India could act as a stepping stone for generating national and subnational level COD estimates. Feasibility studies based on this mobile app need to be undertaken across different regions to develop a VA model, which can be integrated into the public health system for generating mortality statistics in India.

## Supplementary material

10.2196/59937 Multimedia Appendix 1 Data elements of World Health Organization verbal autopsy questionnaire for adults, children, and neonates.

## Abbreviations

ANM: auxiliary nursing midwife
API: application programming interface
ASHA: accredited social health activist
COD: cause of death
ICMR: Indian Council of Medical Research
MCCD: medically certified cause of death
NIRDHDS: National Institute for Research in Digital Health and Data Science
ODK: Open Data Kit
PCVA: physician-coded verbal autopsy
VA: verbal autopsy
WHO: World Health Organization
