# Supplementary material for: Unified Mobile App for Streamlining Verbal Autopsy and Cause of Death Assignment in India: Design and Development Study
Source: JMIR Form Res. 2025 Jan 10;9:e59937. doi: 10.2196/59937 (PMC11755186; doi:10.2196/59937)
Supplement: Multimedia Appendix 1 [file formative-v9-e59937-s001.pdf]

Multimedia Appendix

Multimedia Appendix 1: Data elements of WHO VA Questionnaire for adult, child & neonate

**Adult**	1. VA interviewer<br>2. Preset HIV-Malaria mortality and season<br>3. Information on the respondent and background about the interview<br>4. Information about the deceased and vital registration<br>4.1. Information on the deceased<br>5. Open narrative<br>6. Medical history associated with final illness<br>6.1. History of injuries/accidents<br>6.2. Injuries and accidents detail<br>7. Health history<br>7.1. Duration of illness<br>7.2. General signs and symptoms associated with final illness<br>7.3. Duration of breathing difficulty<br>7.4. Duration of the chest pain<br>7.5. Abdominal pain<br>8. Signs and symptoms associated with pregnancy and women<br>8.1. Questions about possible maternal deaths<br>8.1.1. How did the mother deliver her baby?<br>9. Risk factors<br>10. Health service utilization<br>11. Civil registration number<br>12. Medical certificate of cause of death
**Child**	1. VA interviewer<br>2. Preset HIV-Malaria mortality and season<br>3. Information on the respondent and background about the interview

	4. Information about the deceased and vital registration<br>4.1. Information on the Deceased<br>5. Open narrative<br>6. Medical history associated with final illness<br>6.1. History of injuries/accidents<br>6.2. Injuries and accidents detail<br>7. Health history<br>7.1. Duration of illness<br>7.2. General signs and symptoms associated with final illness<br>7.3. Duration of breathing difficulty<br>7.4. Duration of the chest pain<br>7.5. Abdominal pain<br>8. Child history, signs, and symptoms<br>8.1. Child questions part A<br>8.1.1. Weight (in grams) of the deceased at birth<br>9. Health service utilization<br>10. Civil registration number<br>11. Medical certificate of cause of death
**Neonate**	1. VA interviewer<br>2. Preset HIV-Malaria mortality and season<br>3. Information on the respondent and background about the interview<br>4. Information about the deceased and vital registration<br>4.1. Information on the Deceased<br>5. Open narrative<br>6. Verification of possible stillbirth<br>6.1. History of injuries/accidents<br>6.2. Injuries and accidents detail<br>7. Health history<br>7.1. Duration of illness<br>7.2. General signs and symptoms associated with final illness<br>7.3. Duration of breathing difficulty

	7.4. Neonatal child questions part C<br>8. Neonatal history, signs and symptoms<br>8.1. Neonatal questions part A<br>8.1.1. Weight (in grams) of the deceased at birth<br>8.2. Neonatal child questions part B<br>8.2.1. How was the baby delivered?<br>9. Health service utilization<br>10. Civil registration number<br>11. Medical certificate of cause of death
